# Transcriptomics and metabolomics association analysis revealed the responses of *Gynostemma pentaphyllum* to cadmium

**DOI:** 10.3389/fpls.2023.1265971

**Published:** 2023-10-09

**Authors:** Yunyi Zhou, Lixiang Yao, Xueyan Huang, Ying Li, Chunli Wang, Qinfen Huang, Liying Yu, Chunliu Pan

**Affiliations:** ^1^Guangxi Traditional Chinese Medicine (TCM) Resources General Survey and Data Collection Key Laboratory, the Center for Phylogeny and Evolution of Medicinal Plants, National Center for TCM Inheritance and Innovation, Guangxi Botanical Garden of Medicinal Plants, Nanning, China; ^2^National Engineering Research Center for Southwest Endangered Medicinal Materials Resources Development, Guangxi Botanical Garden of Medicinal Plants, Nanning, China

**Keywords:** *Gynostemma pentaphyllum*, cadmium tolerance, physiological analysis, gene expression, metabolic level

## Abstract

*Gynostemma pentaphyllum* an important medicinal herb, can absorb high amounts of cadmium (Cd) which can lead to excessive Cd contamination during the production of medicines and tea. Hence, it is crucial to investigate the response mechanism of *G. pentaphyllum* under Cd stress to develop varieties with low Cd accumulation and high tolerance. Physiological response analysis, transcriptomics and metabolomics were performed on *G. pentaphyllum* seedlings exposed to Cd stress. Herein, *G. pentaphyllum* seedlings could significantly enhance antioxidant enzyme activities (POD, CAT and APX), proline and polysaccharide content subject to Cd stress. Transcriptomics analysis identified the secondary metabolites, carbohydrate metabolism, amino acid metabolism, lipid metabolism, and signal transduction pathways associated with Cd stress, which mainly involved the XTH, EXP and GST genes. Metabolomics analysis identified 126 differentially expressed metabolites, including citric acid, flavonoid and amino acids metabolites, which were accumulated under Cd stress. Multi-omics integrative analysis unraveled that the phenylpropanoid biosynthesis, starch, and sucrose metabolism, alpha-linolenic acid metabolism, and ABC transporter were significantly enriched at the gene and metabolic levels in response to Cd stress in *G. pentaphyllum*. In conclusion, the genetic regulatory network sheds light on Cd response mechanisms in *G. pentaphyllum*.

## 1 Introduction

Cadmium (Cd) is a naturally occurring heavy metal, and it is a non-essential element for plant growth. In recent years, the increasingly serious Cd contamination in soil has become an important factor restricting sustainable agricultural development and food health and safety in many regions around the world. Cd can readily be absorbed and transported, and it will accumulate in plant tissues. Plants suffer irreversible damage due to the high toxicity and poor degradability of Cd at elevated concentrations. Cd toxicity causes excessive production of reactive oxygen species (ROS), which affects photosynthesis, water balance, gas exchange, and mineral intake, as well as inhibiting plant growth and organ development (Kumar et al., 2023). Various strategies to detoxify Cd have arisen in plants when they are exposed to Cd stress. As a physical barrier, the plant’s cell wall can adsorb and fix Cd by the use of negatively charged substances, and this partially prevents Cd from entering the protoplasts (Meyer et al., 2015; Shi et al., 2015). Some small organic molecules such as phytochelatin, metallothionein and glutahtione in protoplasts are induced to bind and chelate with Cd (Li et al., 2023c). After entering cells, compartmentalization in the plant’s transport mechanism will channel the heavy metals to vacuoles (Shi et al., 2015; Xu et al., 2017). In these organelles, the antioxidant enzymes such as POD, SOD, APX and CAT as well as non-enzymatic antioxidants (including proline, soluble sugars and proteins), are activated to remove excess ROS accumulation (Yuan et al., 2013; Shahid et al., 2017). The plant’s response to Cd stress has been well studied, clearly understanding the physiology of detoxification strategies. Furthermore, a complicated regulatory network involving numerous genes in Cd tolerance has been identified. Consequently, there is an urgent need to unravel the molecular and metabolic regulatory mechanisms underlying the plant’s response to Cd stress.

Recently, there has been significant research on the Cd detoxification molecular mechanisms in plants. The genes involved in the *S*-adenosylmethionine cycle, metal transport, and vacuolar sequestration were found to be regulated differently under Cd stress conditions in maize (Lin et al., 2022). This was also the case for the genes encoding the antioxidant system in rice (Yang et al., 2022). Moreover, the cell wall biosynthesis genes (Casparian strip membrane domain protein (CASP)-like proteins (CSPLs), cell wall protein (CWP), and classical arabinogalactan protein 9-like (CAP9) were identified to play a critical role in Cd detoxification in *Solanum nigrum* (Wang et al., 2022). In addition, some crucial metabolisms that directly protect cells from Cd stress were highlighted. For example, the metabolism of galactose, lipid, and glutathione in buckwheat (Huo et al., 2023), and the ABC transporter, phenylpropanoid biosynthesis and flavonoid biosynthesis pathway in sorghum (Jiao et al., 2023); the unsaturated fatty acids, amino acids (including phenylalanine), nucleotides, sulfur compounds, flavonoids, glutathione and lignin biosynthetic metabolism in *Pistia stratiotes* were also implicated (Wei et al., 2023). The metal-transport genes related to Cd uptake, transport, and detoxification have also been extensively investigated. Such as natural resistance-associated macrophage protein (NRAMP) (Chen et al., 2021), zinc (Zn)-/iron-regulated transporter-like protein (ZIP) (Liu et al., 2019), heavy metal ATPase (HMA) (Qiao et al., 2018) and plant Cd resistance protein (PCR) (Lin et al., 2020). Transcription factors (TFs) have crucial roles in regulating transcription and are important for plants to respond to Cd stress. Recent studies in *Tamarix hispida* have revealed multi-layered transcriptional networks comprising 53 TFs and 54 structural genes, with 341 regulatory relationships predicted, as well as *ThDRE1A*, *ThMYC1* and *ThFEZ*, and modulation of the SOD, CAT and POD activities to scavenge ROS after Cd treatment (Xie et al., 2023). *PvERF15* and *GmWRKY142* were specifically demonstrated to decrease Cd uptake and enhance plant Cd tolerance (Lin et al., 2017; Cai et al., 2020). Hence, it is crucial to construct an accurate molecular regulatory model to identify potential hub genes and metabolites, which is especially important to comprehend the stress response mechanisms in plants.

*Gynostemma pentaphyllum* (Thunb.) Makino, a well-known economically valuable medicinal plant functioning in health care and disease treatment, belongs to the *Gynostemma* genus of the Cueurbitaeeae family and is widely distributed in subtropical regions of East and Southeast Asia (e.g. China, Vietnam, Laos and Malaysia) (Zhang et al., 2019). The leaves of this medicinal plant contain several saponins, polysaccharides, flavonoids, phytosterols and other bioactive ingredients that effectively act as anti-cancer and anti-atherogenic agents as well as affording neuroprotective and hepatoprotective properties (Su et al., 2021). Apart from its use in traditional Chinese medicine, *G. pentaphyllum* leaves are also utilized in tea production (Long et al., 2023). Due to its diverse range of applications, *G. pentaphyllum* has been praised as “southern ginseng” in China. Moreover, *G. pentaphyllum* has a wide range of ecological adaptations and it has a rapid growth rate. These growth advantages make *G. pentaphyllum* highly capable of accumulating heavy metals in contaminated soil, leading to excessive Cd contamination when it is used for tea (Suntararuks et al., 2008). Research has demonstrated that *G. pentaphyllum* plants possess a high ability to absorb Cd, and its Cd concentration is positively correlated with the concentration of the metal in the soil (Nookabkaew et al., 2016). Currently, the Cd tolerance and accumulation characteristics have been studied in *Gynostemma* plants, and it was found that when they are grown in the presence of high Cd concentrations, this reduced the growth, biomass, and chlorophyll content of three different *Gynostemma* species (Li et al., 2022b). These studies indicated that *Gynostemma* plants have specific tolerance and accumulation characteristics for Cd. Nevertheless, there is still a lack of accurate understanding regarding the Cd tolerance and accumulation characteristics in *G. pentaphyllum*. Currently, there is limited knowledge regarding the molecular mechanisms underlying the response of *G. pentaphyllum* to Cd. In the current study, *G. pentaphyllum* seedlings were chosen and their physiological, genetic and metabolic responses were investigated when subjected to various levels of Cd treatment. This study aimed to investigate the main genes, metabolites and key metabolic pathways associated with *G. pentaphyllum* in response to different levels of Cd stress. This study uncovers how *G. pentaphyllum* responds to Cd stress at both the physiological and molecular levels. It provides insights into the *G. pentaphyllum* Cd response mechanisms, which can potentially guide the future selection and cultivation of plant varieties with low Cd accumulation and high tolerance varieties.

## 2 Materials and methods

### 2.1 Plant material

*G. pentaphyllum* was obtained from the Guangxi Botanical Garden of Medicinal Plants (Nanning, Guangxi, China). The seeds were germinated on moist perlite, after which seedlings of 3~5 leaf stage were selected and cultivated in hydroponic boxes with the Japanese Yamzaki formula solution (pH 6.0) (Li et al., 2023b). All plants were cultivated at a constant temperature of 25 ± 2°C, with a photoperiod of 14 h/10 h (day/night) and light intensity of 100 μmol m^-2^ s^-1^). After 7 days of culture, the seedlings were subjected to a hydroponic medium containing different concentrations of CdCl_2_ (0, 25, 50, 100, 150 and 200 μM) for 7 days, and seedlings grown in Cd-free hydroponic medium were used as controls (Li et al., 2022b). The *G. pentaphyllum* plants were then collected and assessed for height and root length. Fresh leaf samples were acquired for the assessment of physiological indexes as well as transcriptomics and metabolomics analysis. All samples were ground in liquid nitrogen by using a JXFSTPRP-64L grinding instrument (Shanghai, China) and stored at -80°C.

### 2.2 Determination of physiological indexes

The enzyme activities of superoxide dismutase (SOD), peroxidase (POD), catalase (CAT), L-ascorbate peroxidase (APX), as well as the contents of malondialdehyde (MDA) and proline were assessed and analyzed utilizing the methods as previously described (Pan et al., 2023). Dithizone (DTZ) staining was employed to evaluate Cd localization at the cellular levels as previously described (He et al., 2013). Periodic acid-Schiff (PAS) staining was employed using the Periodic Acid Schiff (PAS) Stain Kit (Beijing Solarbio Technology) by following the manufacturer’s instructions. A Zeiss Axioscope 5 microscopy (Zeiss, Germany) was utilized to capture the images. Each experiment utilized three replicates of each treatment regimen.

### 2.3 High throughput transcriptomics analysis

Fresh leaf samples of Cd0 (CK), Cd25 (LC) and Cd100 (HC) were used in transcriptomics profiling analysis. The total RNA of nine samples from the three conditions was extracted using a Trizol reagent kit (Invitrogen, Carlsbad, CA, USA) according to the manufacturer’s instructions. The purity, concentration and integrity of the RNA samples were assessed. The cDNA libraries were prepared using the NEBNext Ultra RNA Library Prep Kit for Illumina (NEB #7530, New England Biolabs, Ipswich, MA, USA). Finally, the cDNA library was sequenced using the Illumina Novaseq 6000 platform in the paired-end mode by Gene Denovo Biotechnology Co., Ltd (Guangzhou, China).

Following the sequencing process, the raw sequence data underwent filtration in order to eliminate low-quality reads (reads with a mass value below 10 and constituting more than 50% of the total bases) by FASTP v0.18.0. The short reads alignment tool Bowtie2 v2.2.8 was used for mapping reads to ribosome RNA (rRNA) database, and the rRNA mapped reads were removed. Then, the clean reads were mapped to the *G. pentaphyllum* reference genome (PRJNA720501, https://www.ncbi.nlm.nih.gov/search/all/?term=PRJNA720501) with HISAT2 v2.2.4. The fragments per kilobase of transcript per million mapped reads (FPKM) for all transcripts were quantified using Trapnell’s method (Trapnell et al., 2010). The differential expressed genes (DEGs) were calculated by using the NOISeq method, employing a |log2 (fold change) |>2 and a false discovery rate (FDR) <0.05 (Tarazona et al., 2011). Gene ontology (GO) enrichment analysis was conducted with the GO database (http://www.geneontology.org/), while KEGG pathway enrichment analysis was conducted using the KEGG database (http://www.kegg.jp/).

### 2.4 Metabonomics profiling analysis

The fresh leaf samples of Cd0 (CK), Cd25 (LC), and Cd100 (HC) were incubated overnight at 4 °C, using 1.0 mL 70% aqueous methanol. The extracts were absorbed and filtered through an SPE cartridge and microporous membrane (0.22 μm pore size) before LC-MS analysis. The metabolomic analysis was conducted utilizing an ultra-performance liquid chromatography-tandem mass spectrometry (UPLC-MS) system. The chromatographic separation was achieved using a Waters C18 column, employing mobile phase A (0.04% acetic acid in water) and mobile phase B (0.05% acetic acid in acetonitrile) and operating at 40°C. Solvent gradient changes were linear for all the steps and these were: 95:5 Phase A/Phase B at 0 min, 5:95 Phase A/Phase B at 11.0 min, 5:95 Phase A/Phase B at 12.0 min, 95:5 Phase A/Phase B at 12.1 min and 95:5 Phase A/Phase B at 15.0 min. The flow rate was 0.4 mL/min, and the injection volume was 2 μL. An electrospray ionization (ESI) mode was employed to acquire the high-resolution mass spectra (HRMS), while operating in the positive ion mode. Further data processing was performed using Analyst 1.6.1 software. Metabolite information was identified by conducting searches in both internal and public databases (MassBank, KNApSAcK, HMDB, MoTo DB, and METLIN). PCA and OPLS-DA were utilized to identify significantly different metabolite levels (p-value < 0.05). Differentially altered metabolites (DAMs) were designated as a log2 fold change (FC) ≥ 2 and FC ≤ 0.5, along with variable importance in projection (VIP) scores > 1. The identified metabolites underwent metabolic pathways analysis using the KEGG database and MetaboAnalyst 4.0 (http://www.metaboanalyst.ca).

### 2.5 Correlation network analysis of transcriptomics and metabolomics data

Correlation network analysis was utilized to generate and analyze the association characteristics between metabolites and genes. The correlation characteristics between genes and metabolites were obtained using the KEGG pathway shared with genes and metabolites. Bidirectional orthogonal projections to latent structures (O2PLS) were employed to analyze both gene expression and metabolite abundance, and the best models derived from this analysis were derived from integration analysis. Gene-metabolite pairs were ranked by absolute correlation coefficients, which were calculated for gene expression and metabolite abundance. The resulting networks were visualized using the Cytoscape version 3.7.2 software package.

### 2.6 Quantitative real-time PCR (qRT-PCR) analysis

Fourteen DEGs were selected for qRT-PCR analysis and actin was used as an internal reference gene. The primers used were based on the transcript sequences using Primer 5.0 (**Table S6**). The qRT-PCR was performed using the LightCycler 96 instrument (Roche, Basel, Switzerland). The 2^–ΔΔCt^ method was employed to calculate the relative expression levels of the genes (Livak and Schmittgen, 2001). When the transcriptomic and qRT-PCR data were combined, the candidate genes appeared to show similar expression patterns (**Figure S7**).

### 2.7 Statistical analysis

Statistical analyses were conducted by using SPSS v 26.0 software. All values were expressed as the means ± standard deviations. All the data were tested using one-way ANOVA with Duncan’s multiple-range test. The p < 0.05 was considered to be statistically significant. The graphs were drawn using GraphPad Prism 8.

## 3 Results

### 3.1 Effect of Cd on the growth changes in *Gynostemma pentaphyllum*

**Figure 1:**
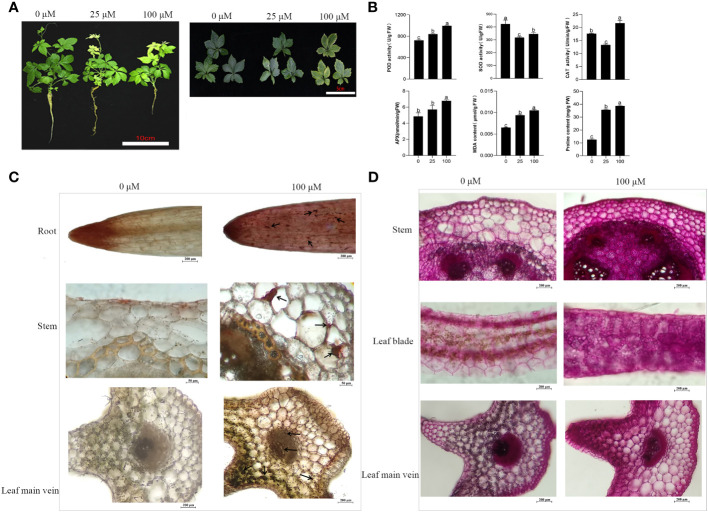
Effects of Cd stress in *G. pentaphyllum* seedlings with different concentrations. **(A)** Typical images of the seedlings. **(B)** Antioxidant enzymes (POD, SOD, APX, CAT) activity and MDA, proline content. **(C)** Cd location in the root, stem and leaf. Arrows show the Cd-dithizone precipitates. **(D)** Cell polysaccharides micrographs of stem and leaf. The bars with different lowercase letters represent significant differences based on one-way ANOVA with Duncan’s multiple range test (p < 0.05).

With increasing the Cd concentrations, the average root lengths of the Cd treatment groups were significantly reduced by 10.33%~40.96% compared to that of the control group, except for the Cd5 group (**Figure S1A**). In addition, with the increase of Cd concentrations, the height of plants also exhibited a notable reduction (**Figure S1B**). The growth of seedlings displayed visible poisoning symptoms and withered in the Cd200 (200 μM) group. The seedlings treated with Cd25 and Cd100 showed the minimum reduction and significant inhibitory in root length and height, respectively (**Figure S1**). These also exhibited different growth behavior (**Figure 1A**), and therefore these two concentrations were chosen for further analysis.

The activities of POD and APX, as well as the concentrations of MDA and proline content, showed a gradual trend with increased Cd stress (**Figure 1B**). In contrast, there was a notable and consistent decline in SOD activity when compared to the Cd0 group. In addition, the activity CAT was reduced by 24.53% in the Cd 50 group and increased by 22.64% compared with that of the Cd0 group (**Figure 1B**). Cd-dithizone precipitates were observed in the roots, stems and leaves (**Figure 1C**). Obvious Cd-dithizone precipitates were also observed in the root tips. Cross-section analysis of the stems showed Cd was mainly located in parenchymatous cells of the cortex. In the leaves, Cd-dithizone precipitates were observed mainly in parenchymatous cells as well as the phloems and xylems of the main veins. However, in the Cd100 group, bright pink polysaccharide staining was seen in the stems and leaves when they were compared with the Cd0 group (**Figure 1D**). All these results indicated that the *G. pentaphyllum* seedlings could efficiently activate the oxidative enzymes, proline content, Cd transport, and polysaccharides when response to Cd stress.

### 3.2 Transcriptomics analysis

**Figure 2:**
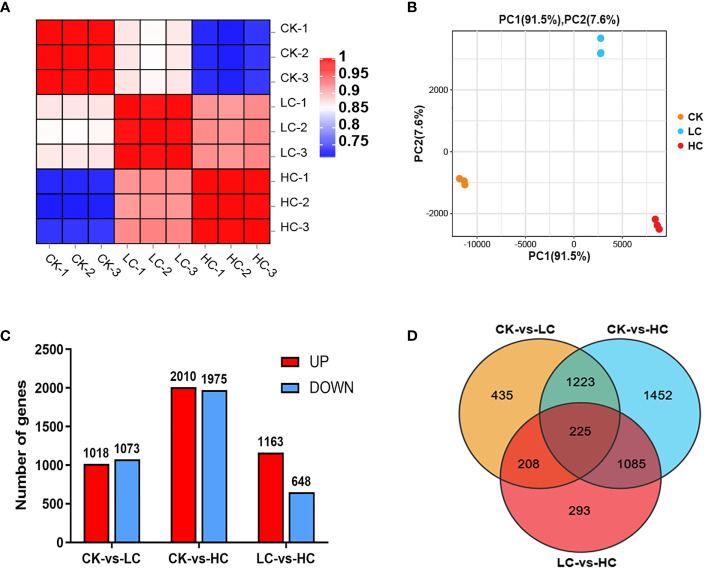
Transcriptome data of Cd stress in *G. pentaphyllum* seedlings among the different treatment groups. **(A)** The Pearson correlation coefficient analysis. **(B)** The PCA analysis. **(C)** Analysis of DEGs number. **(D)** Venn graph of DEGs.

On average, a total of 42160383 clean reads were obtained from each of the samples (**Table S1**). The GC content, Q20, and Q30 values of all clear reads were above 45.55%, 97.55%, and 93.16%, respectively, confirming the high reliability of the sequencing outcomes. The clean reads from all samples had a mapping rate of 85.16~86.67% when compared to the reference genome sequence, resulting in the discovery of 25,656 genes. The Pearson correlation coefficient analysis provided evidence of the biological consistency (**Figure 2A**). In addition, the PCA analysis demonstrated discernible differences in the expression of gene clusters between the control group (Cd0, CK) and the different Cd treatments (Cd25, LC and Cd100, HC) were distinguishable (**Figure 2B**). 2091 (1018 up- and 1073 down-regulated), 3985 (2010 up- and 1975 down-regulated), and 1811 (1163 up- and 648 down-regulated) DEGs were identified by comparing CK vs LC, CK vs HC, and LC vs HC, respectively (**Figure 2C**). In all comparison groups, a comprehensive set of 4921 DEGs were screened (**Figure 2D** and **Table S2**). Among these, 225 genes were screened that were commonly expressed across all groups (**Figure 2D**), manifesting that *G. pentaphyllum* activated the expression levels of these genes to cope with varying levels of Cd stress.

### 3.3 GO and KEGG pathway analysis of DEGs

The GO enrichment analysis confirmed the impact of Cd stress on specific biological functions with the top 20 GO enriched terms (**Figure S2** and **Table S3**). The oxidoreductase activity (GO:0016491), tetrapyrrole binding (GO:0046906) and photosystem (GO:0009521) with enriched GO terms were found mainly in CK vs LC. The protein kinase activity (GO:0004672), protein phosphorylation (GO:0006468) and photosystem (GO:0009521) with enriched GO terms were found mainly in CK vs HC. The DEGs were notably enriched in protein kinase activity (GO:0004672), protein phosphorylation (GO:0006468), and response to chitin (GO:0010200) with LC vs HC comparison. GO analysis indicated that GO terms such as oxidoreductase activity, protein kinase activity and protein phosphorylation could effectively enhance the Cd tolerance of *G. pentaphyllum*.

The KEGG pathway enrichment analysis confirmed the impact of Cd stress on specific biological pathways. A higher number of DEGs were significantly assigned to enrich the metabolic pathways and biosynthesis of secondary metabolites in CK vs LC, CK vs HC, and LC vs HC pairwise groups (**Figure S3**). In the CK vs LC comparison, several pathways, including phenylpropanoid biosynthesis, plant hormone signal transduction, photosynthesis, starch and sucrose metabolism, alpha-linolenic acid metabolism and flavonoid biosynthesis, showed significant enrichment. The significant enrichment pathways in the comparison between CK and HC included phenylpropanoid biosynthesis, photosynthesis, porphyrin metabolism, starch and sucrose metabolism, MAPK signaling pathway and biosynthesis of various alkaloids. The phenylpropanoid biosynthesis, plant-pathogen interaction, biosynthesis of various alkaloids, MAPK signaling pathway, phenylalanine metabolism and flavonoid biosynthesis were significantly enriched pathways in LC vs HC. According to the KEGG analysis, the response of *G. pentaphyllum* to Cd resulted in the regulation of various pathways, including the biosynthesis of other secondary metabolites, carbohydrate metabolism, amino acid metabolism, lipid metabolism, and signal transduction.

**Figure 3:**
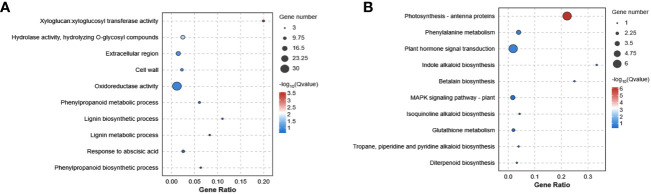
The enrichment analysis of GO **(A)** and KEGG **(B)** functional pathways of Cd stress in *G. pentaphyllum*.

In addition, the detailed functions of the common 225 genes from the three comparison groups were investigated. The xyloglucosyl transferase activity, hydrolase activity, extracellular region, cell wall, oxidoreductase activity and phenylpropanoid metabolic process were mainly enriched in GO terms (**Figure 3A**). Additionally, these DEGs were categorized based on their involvement in KEGG pathways analysis. The photosynthesis, phenylalanine metabolism, plant hormone signal transduction, indole alkaloid biosynthesis, betalain biosynthesis and MAPK signaling pathway were the most enriched pathways (**Figure 3B**). These findings indicated that Cd stress had a substantial impact on multiple physiological processes, notably affecting amino acid metabolism, carbon metabolism, signal transduction, and secondary metabolic systems.

### 3.4 Analysis of genes involved in Cd response

To further elucidate the role of Cd response for 225 genes, the ABC transporters, cell wall, phenylpropanoid biosynthesis, glutathione metabolism, photosynthesis and TFs were selected for analysis (**Table 1**). There were two genes related to ABC transporters that appeared to be important. ABCG23 (ABC transporter G family member 23) was up-regulated in CK vs HC and LC vs HC, and ABCG8 (ABC transporter family member 8) was down-regulated in all three comparison groups. Three down-regulated xyloglucan endotransglucosylase/hydrolase (XTH31, XTH7 and XTH9) and one down-regulated expansin (EXPA10) involved in the cell wall in the three comparison groups, and two up-regulated XTH23 and XTH2 in CK vs HC and LC vs HC, respectively. Notably, two genes associated with glutathione S-transferase (GST) and related to glutathione metabolism were up-regulated, while six chlorophyll a-b binding protein genes involved in photosynthesis were down-regulated. As many as 12 TFs were also found, underscoring their importance in mediating the Cd response and transportation processes. NAC, ERF4, MYB39, CPRF1 and HSFC1 genes were up-regulated, and bHLH70, COL16, and RL1 genes were down-regulated in three comparison groups. All these *G. pentaphyllum* genes were likely linked to the ability of this species to withstand the response to Cd stress.

**Table 1:** List of DEGs possibly involved in Cd response of *G. pentaphyllum*.

Unigene ID	Gene annotation	Gene name	Log2 of fold change		
			CK-vs-LC	CK-vs-HC	LC-vs-HC
ABC transporters					
mikado.CM035879.1G2866	ABC transporter G family member 23	ABCG23	-1.20	1.27	2.48
mikado.CM035879.1G3181	ABC transporter G family member 8	ABCG8	-1.08	-2.13	-1.05
cell wall					
mikado.CM035879.1G2690	xyloglucan endotransglucosylase/hydrolase protein 31-like	XTH31	-1.34	-2.51	-1.17
mikado.CM035884.1G2617	xyloglucan endotransglucosylase/hydrolase protein 23	XTH23	-1.98	1.66	3.64
mikado.CM035889.1G1856	xyloglucan endotransglucosylase/hydrolase protein 7	XTH7	-1.68	-2.93	-1.25
mikado.CM035889.1G278	xyloglucan endotransglucosylase/hydrolase protein 9-like	XTH9	-1.03	-2.21	-1.18
mikado.CM035889.1G70	xyloglucan endotransglucosylase/hydrolase 2-like	XTH2	-1.04	2.78	3.82
mikado.CM035882.1G905	expansin-A10-like	EXPA10	-1.73	-2.92	-1.19
Phenylpropanoid biosynthesis					
mikado.CM035879.1G2678	phenylalanine ammonia-lyase-like	PAL	-2.08	-3.99	-1.91
mikado.CM035882.1G2411	phenylalanine ammonia-lyase-like	PAL	-1.52	1.31	2.83
mikado.CM035879.1G2959	peroxidase 2-like	PER2	-4.08	-2.46	1.62
mikado.JAHXMR010000013.1G34	peroxidase 40-like	PER40	-1.31	-2.37	-1.06
Glutathione metabolism					
mikado.CM035881.1G108	glutathione *S*-transferase U9-like	GSTU9	1.10	3.08	1.98
mikado.CM035883.1G1246	glutathione *S*-transferase	GST	2.54	4.24	1.70
Photosynthesis					
mikado.CM035881.1G136	chlorophyll a-b binding protein P4	LHCA-P4	-1.87	-2.91	-1.05
mikado.CM035881.1G1201	chlorophyll a-b binding protein of LHCII type 1-like	CAB	-3.58	-5.41	-1.83
mikado.CM035881.1G1203	chlorophyll a-b binding protein of LHCII type 1-like	CAB	-3.81	-5.01	-1.20
mikado.CM035881.1G1204	chlorophyll a-b binding protein of LHCII type 1-like	CAB	-3.82	-5.16	-1.34
mikado.CM035881.1G1189	chlorophyll a-b binding protein 3	LHCB3	-2.90	-4.09	-1.19
mikado.CM035883.1G565	chlorophyll a-b binding protein 3	LHCB3	-1.41	-2.58	-1.17
Transcription factor					
mikado.CM035879.1G1883	NAC transcription factor 29-like	NAC	1.03	2.63	1.61
mikado.CM035889.1G563	NAC domain-containing protein 35-like	NAC	1.32	3.76	2.44
mikado.CM035881.1G1043	ethylene-responsive transcription factor 4	ERF4	1.02	2.30	1.28
mikado.CM035882.1G1403	zinc finger protein ZAT12-like	ZAT12	-1.09	1.35	2.44
mikado.CM035882.1G3397	transcription factor MYB39	MYB39	1.53	2.94	1.41
mikado.CM035885.1G2092	transcription factor MYB44-like	MYB44	-1.26	1.21	2.47
mikado.CM035887.1G539	transcription factor MYB44-like	MYB44	-1.49	1.19	2.68
mikado.CM035885.1G1089	transcription factor bHLH67	bHLH70	-1.67	-3.20	-1.54
mikado.CM035885.1G894	transcriptional activator TAF-1-like	CPRF1	2.08	3.10	1.02
mikado.CM035886.1G1570	heat stress transcription factor C-1-like	HSFC1	1.03	2.07	1.03
mikado.CM035888.1G2676	zinc finger protein CONSTANS-LIKE 16-like	COL16	-1.66	-2.78	-1.12
mikado.CM035889.1G766	protein RADIALIS-like 1	RL1	-4.09	-13.54	-9.45

### 3.5 Metabonomics analysis

**Figure 4:**
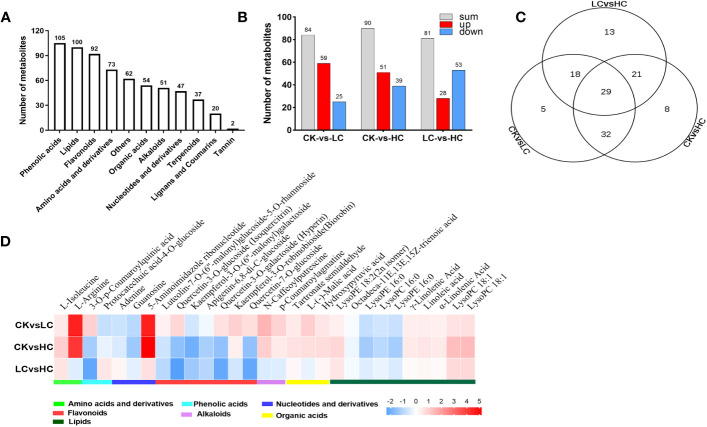
Merabolites analysis of *G. pentaphyllum* responding to Cd stress. **(A)** The number of different metabolites types. **(B)** Analysis of DAMs number. **(C)** Venn graph of DAMs. **(D)** Heatmaps of DAMs compared between different groups.

To explore the response of *G. pentaphyllum* under different Cd stresses, metabolomics analysis was performed using data obtained from UPLC-MS/MS. The control (Cd0, CK) and Cd-treated groups (Cd25, LC and Cd100, HC) were distinguished by PCA and OPLS-DA score plots (**Figure S4**). In total, 643 metabolites were screened (**Figure 4A** and **Table S4**), of which 126 DAMs were identified in all groups (**Figure 4B** and **Table S5**). In the CK vs LC, CK vs HC, and LC vs HC comparisons, there were 84, 90 and 81 DAMs, respectively (**Figure 4B**). The number of DAMs was notably higher in CK vs HC than in other combinations, indicating that there was a specific impact on the stimulation of certain metabolites by a high concentration of Cd. By performing KEGG enrichment analysis (**Figure S5**), it was observed that pathways involved with pyruvate metabolism, citrate cycle (TCA cycle), glyoxylate and dicarboxylate metabolism were significantly enriched due to DAMs in pairwise CK vs LC. Arginine and proline metabolism, and the TCA cycle were significantly enriched in pairwise CK vs HC. Flavone and flavonol biosynthesis, valine, leucine and isoleucine biosynthesis were significantly enriched in pairwise LC vs HC. The results strongly implied that these metabolites were essential for *G. pentaphyllum* response to Cd stress.

### 3.6 Analysis of metabolites involved in Cd response

The Venn diagram analysis showed that 29 DAMs were found to be affected by different Cd concentration treatments (**Figure 4C** and **Table S4**). These metabolites showed significant induction with increasing Cd intensity in CK vs LC, CK vs HC, and LC vs HC, including 10 lipids, 7 flavonoids, 3 nucleotides and derivatives, 3 organic acids, 2 amino acids and derivatives, 2 phenolic acids and 2 alkaloids (**Figure 4D**). Based on the log_2_ fold change values, it was evident that 15 DAMs were up-regulated in CK vs LC and CK vs HC, and these included L-isoleucine, L-arginine, 5-aminoimidazole ribonucleotide, and kaempferol-3-O-robinobioside (biorobin). The down-regulated DAMs in CK vs HC and LC vs HC were protocatechuic acid-4-O-glucoside, adenine, guanosine and kaempferol-3-O-(6’’-malonyl) galactoside. Therefore, these DAMs can serve as potential candidate markers for the response of *G. pentaphyllum* to Cd stress and act as stimuli for distinguishing exposure to various Cd concentrations.

### 3.7 Integrated transcriptomics and metabolomics analysis

**Figure 5:**
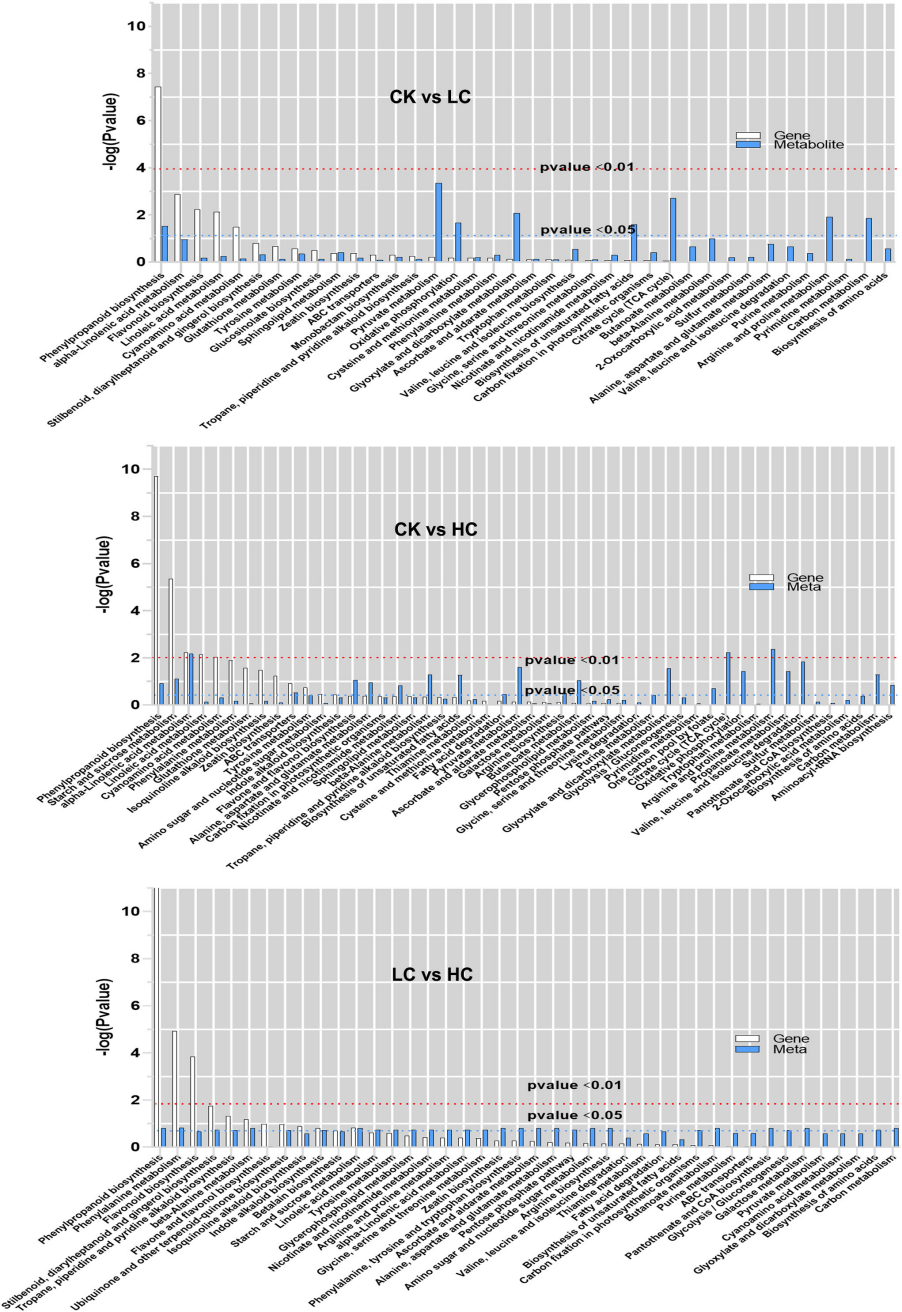
KEGG pathway enrichment analysis of DEGs and DAMs in transcriptomic and metabolomics. The abscissa represents metabolic pathways, and the ordinate represents the enriched P-value of DEGs (white) and DAMs (blue), which is represented by -log (p-value) using a threshold of p < 0.01 and p < 0.05.

The histogram depicted the degree of KEGG pathway enrichment when considering both DEGs and DAMs simultaneously (**Figure 5**). 205 DEGs and 87 DAMs were enriched to 38 metabolic pathways in CK vs LC, 532 DEGs, and 128 DAMs were enriched to 50 metabolic pathways in CK vs HC, and 203 DEGs, and 82 DAMs were enriched to 42 metabolic pathways in LC vs HC. Interestingly, it was intriguing to observe that DEGs and DAMs in CK vs LC were simultaneously significantly enriched in the phenylpropanoid biosynthesis pathway, which was stimulated by low concentrations of Cd. The phenylpropanoid biosynthesis pathway, starch and sucrose metabolism, alpha-linolenic acid metabolism and ABC transporters were found significantly enriched for DEGs and DAMs simultaneously in CK vs HC. The results suggested that even under high Cd stress, these metabolic pathways were still active and they were stimulated simultaneously to the ABC transporter. The phenylpropanoid biosynthesis pathway, phenylalanine metabolism, beta-alanine metabolism and starch and sucrose metabolism were significantly enriched by DEGs and DAMs simultaneously in LC vs HC, and this showed that these pathways were promoted simultaneously by Cd stress. The phenylpropanoid biosynthesis pathway, starch and sucrose metabolism, alpha-linolenic acid metabolism and ABC transporter were the vital pathways for Cd response of *G. pentaphyllum*.

#### 3.7.1 Potential candidate DEGs and DAMs involved in alpha-linolenic acid metabolism

**Figure 6:**
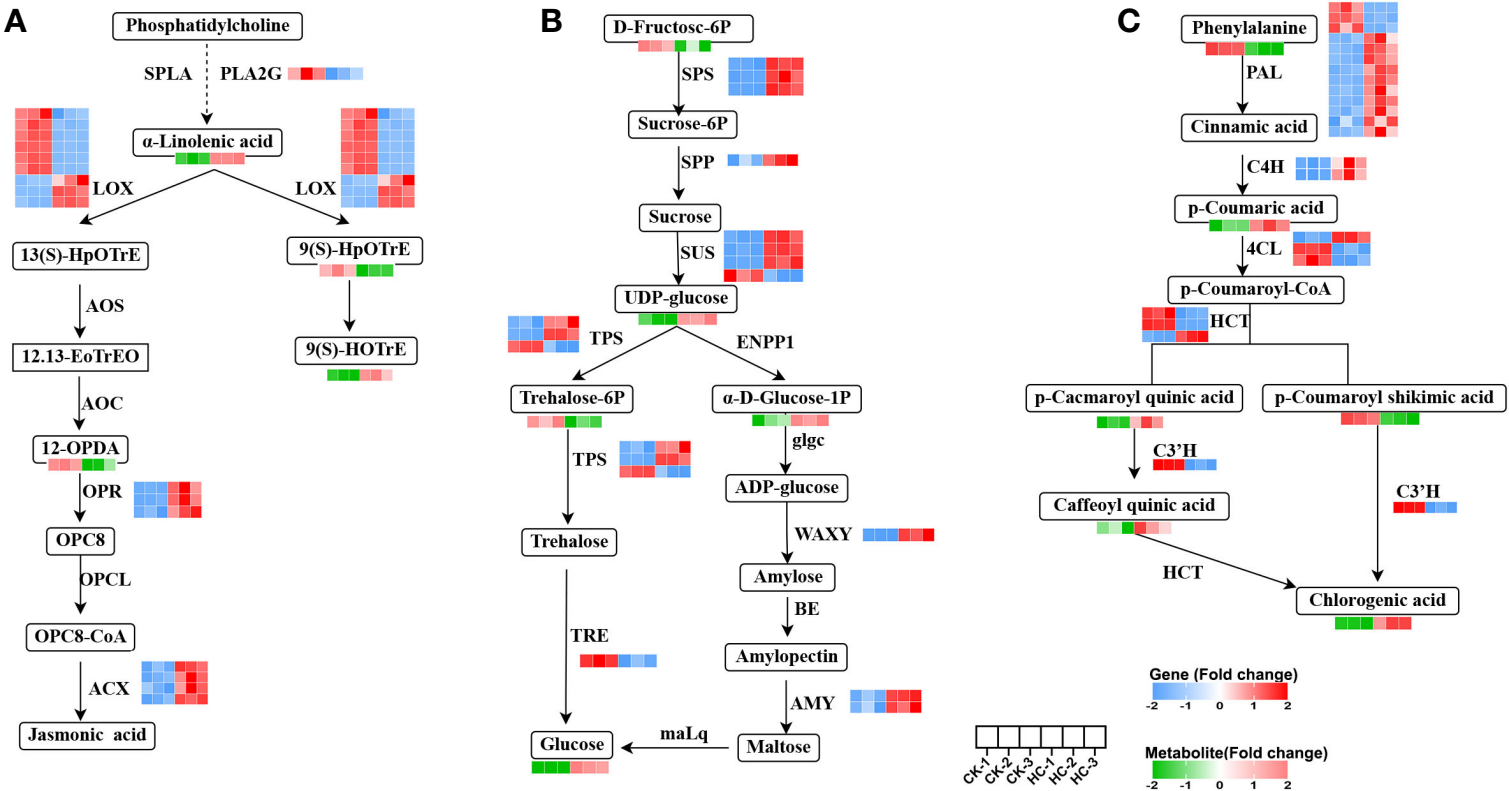
Changes of DEGs and DAMs involved in main metabolic pathways in *G. pentaphyllum* with Cd stress. **(A)** Alpha-linolenic acid metabolism. **(B)** Starch and sucrose metabolism. **(C)** Phenylpropanoid biosynthesis pathway.

The 17 genes with significant differences in linolenic acid metabolism were: one PLA2G (secretory phospholipase A2), nine LOX (lipoxygenase), three OPR (12-oxophytodienoic acid reductase), four ACX (acyl-CoA oxidase) genes; and the four metabolites, a-linolenic acid, 9(S)-HpOTrE, 9(S)-HOTrE and 12-OPDA (**Figure 6A**). Four ACX, three LOX, three OPR genes, and two metabolites (a-linolenic acid, 9(S)-HOTrE) were up-regulated, while six LOX, one PLA2G gene and two metabolites (9(S)-HpOTrE, 12-OPDA) were down-regulated in Cd treatment, compared to the CK. These findings highlighted ACX, LOX, OPR, and PLA2G as pivotal genes in the alpha-linolenic acid metabolism pathway, which crucially responded to Cd stress. Changes in their expression levels facilitated the synthesis of the crucial metabolites, especially the biosynthesis of jasmonic acid (JA), which is involved in numerous stress responses in plants (Wasternack and Strnad, 2018).

#### 3.7.2 Potential candidate DEGs and DAMs involved in starch and sucrose metabolism

By integrating the analysis of genes and metabolites, 15 DEGs and 5 DAMs were found to be related to starch and sucrose metabolism (**Figure 6B**). Of these, three SPS (sucrose-phosphate synthase), three SUS (sucrose synthase), two TPS (alpha, alpha-trehalose-phosphate synthase), two AMY (alpha-amylase), one WAXY (granule-bound starch synthase) genes and three metabolites (glucose, alpha-D-glucose-1-phosphate[α-D-Glucose-1P], uridine 5’-diphospho-D-glucose[UDP-glucose]) were up-regulated, while one SUS, one TPS, one TRE (trehalase) genes and two metabolite (D-fructose-6P and trehalose 6-P) were down-regulated in Cd treatment, when compared to the CK. SPS, SUS, TPS, AMY, WAXY, and TRE were identified as key genes in the starch and sucrose metabolism pathway, which significantly responded to Cd stress. The synthesis of the glucose metabolite was triggered by their changes in expression levels.

#### 3.7.3 Potential candidate DEGs and DAMs involved in phenylpropanoid biosynthesis

The 22 genes with significant differences in phenylpropanoid biosynthesis were: thirteen PAL (phenylalanine ammonia-lyase), two C4H (trans-cinnamate 4-monooxygenase), three 4CL (4-coumarate-CoA ligase), three HCT (shikimate O-hydroxy cinnamoyl transferase), one C3H (p-coumaroyl shikimate 3-hydroxylase) genes. There were also six metabolites (phenylalanine, p-coumaric acid, p-coumaroyl-shikimic acid, p-coumaroyl-quinic acid, caffeoylquinic acid and chlorogenic acid) with significant differences (**Figure 6C**). Nine PAL, two C4H, one 4CL, one HCT gene and three metabolites (p-Coumaric acid, 5-O-p-coumaroylquinic acid, and chlorogenic acid) were up-regulated, while three PAL, two C4H, five 4CL, three HCT, one C3H genes and two metabolites (phenylalanine and p-coumaroyl-shikimic acid) were down-regulated after Cd treatment, when compared to the CK. These results remarkably suggested that Cd stress exerted a substantial influence on the regulation of gene expression and metabolite accumulation in phenylpropanoid biosynthetic pathways of *G. pentaphyllum*.

#### 3.7.4 Potential candidate DEGs and DAMs involved in ABC transporters

**Figure 7:**
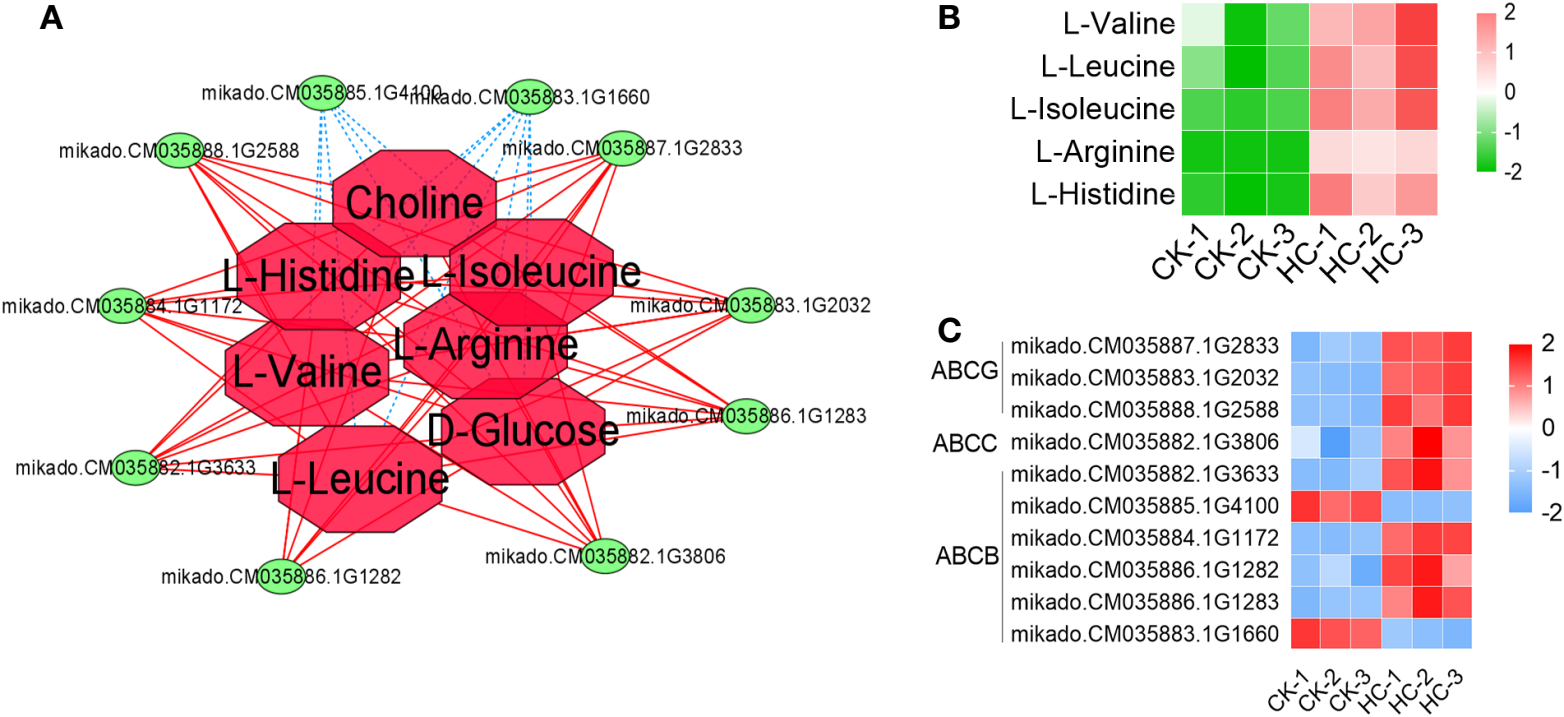
Changes of DEGs and DAMs involved in the pathway of ABC transporter in *G. pentaphyllum* with Cd stress. **(A)** The network analysis of DEGs and DAMs. **(B)** Heatmaps of DAMs. **(C)** Heatmaps of DEGs.

A total of 10 ABC transporter family member genes and 5 DAMs were involved in the ABC transporter (**Figure 7A**). Eight genes and five metabolites were considerably up-regulated, while two genes were significantly down-regulated after Cd treatment, when compared to the CK (**Figures 7B, C**). These results suggested that the ABC transporter family member gene expression and the accumulation of amino acids within the ABC transporter played important roles in *G. pentaphyllum* response to Cd stress.

#### 3.7.5 Potential candidate TFs involved in Cd response

**Figure 8:**
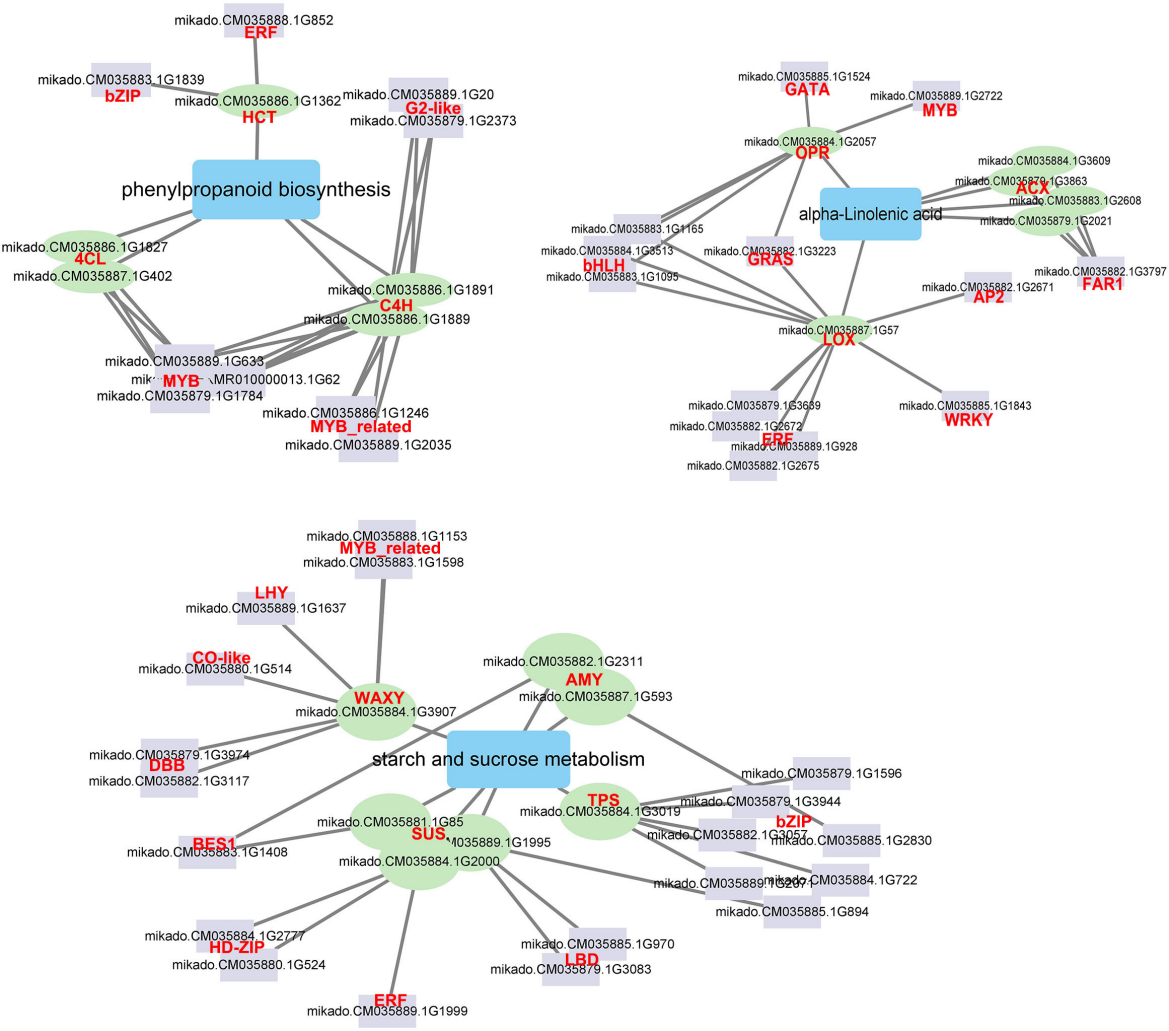
The correlation network of TFs and DEGs involved in phenylpropanoid biosynthesis, starch and sucrose metabolism, and alpha-linolenic acid metabolism. The purple rectangle represents TFs, the green circle represents DEGs; the blue rectangle represents metabolic pathway; the line represents interaction with correlation.

A network was created depicting the TFs interactions with the key pathway genes to understand the detailed molecular mechanism in response to Cd stress (**Figure 8**). Five TF families interacted with the phenylpropanoid biosynthesis genes. The ERF and bZIP TFsinteracted with one HCT gene, and three MYB, two MYB_related, and two G2-like TFs interacted with four phenylpropanoid biosynthesis genes (C4H and 4CL), respectively. Two AMY, one WAXY, one TPS and three SUS genes, which have been demonstrated as hub genes for starch and sucrose metabolism, were also found to be regulated by 10 TF families such as MYB_related, bZIP, BES1 and ERF. Eight TF families interacted with alpha-linolenic acid genes. Four ERF, three bHLH, one WRKY, one GRAS and one AP2 TF interacted with the LOX gene, the FAR TF interacted with four ACX genes, and GATA and MYB TF interacted with the OPR gene, respectively. There were 12 TF families related to MYB, MYB_related, ERF, GRAS, bZIP, WRKY and bHLH which showed significantly different expression levels after Cd treatment (**Figure S6**). The network analysis indicated that TFs such as MYB, MYB_related, ERF, GRAS, bZIP, WRKY, and bHLH can effectively impact genes associated with phenylpropanoid biosynthesis, starch and sucrose metabolism and alpha-linolenic acid metabolism and improve the Cd response of *G. pentaphyllum*.

## 4 Discussion

### 4.1 The effects of Cd on the progression and physiology of *Gynostemma pentaphyllum*

Cd, which is classified as a non-essential heavy metal, can inhibitplant growth which is demonstrated by both physiological and biochemical indicators (Rizwan et al., 2017; Gul et al., 2021). In this study, Cd treatment showed a dwarf phenotype on *G. pentaphyllum*, underscoring the detrimental effects of Cd, and was consistent with previous studies (Li et al., 2022b). Prior research had demonstrated that treatment with Cd in sorghum and wheat seedlings had toxic effects and this was concentration-dependent (Jiao et al., 2023; Zhang et al., 2023). Cd treatment with low concentrations (1.6 mg/L^-1^) manifested the best stimulatory growth when compared to high concentrations (6.5 mg/L^-1^) in young peppermint plants (Wang et al., 2023). In this study, *G. pentaphyllum* root growth responds to Cd with stimulation at low doses and inhibition at high doses, as evidenced by the more pronounced growth inhibition in seedlings treated with higher Cd concentrations compared to those treated with lower concentrations (**Figures 1A**, **S1**). Previous studies have verified that the Cd accumulation of leaves is higher than that in roots of *Calotropis gigantean* in Cd-polluted environments (Yang et al., 2022). Our results show that Cd mainly accumulates in the root tips, as well as in different structural tissues such as the stems and leaves (**Figure 1**). Biomass of *G. pentaphyllum* in the aboveground parts is far greater than that of underground portions, and Cd is suggested to be mainly accumulated in the former.

To cope with the cytotoxicity of Cd, plants have developed multiple tolerance mechanisms. The plant’s antioxidant defense system, which includes a variety of antioxidant enzymes (POD, SOD, APX and CAT), as well as non-enzymatic antioxidants (proline, soluble sugars and proteins), plays a critical role in scavenging ROS stress generated due to Cd (Rizwan et al., 2016; Rizwan et al., 2017; Li et al., 2023c). Cellulosic polysaccharides, such as pectin, cellulose and hemicellulose, have significant functions in the binding and accumulation of Cd under Cd stress conditions (Wan and Zhang, 2012; Meyer et al., 2015). The activities of POD, CAT, APX, and proline content exhibited a consistent increase with rising concentrations of Cd (**Figure 1B**). Furthermore, the main components of the cell wall, which are the polysaccharides, were seen to accumulate under Cd stress (**Figure 1D**). This suggests that activation of antioxidant system and e stimulation of metal binding-related cell wall polysaccharides are the important physiological characteristics of *G. pentaphyllum* in response to Cd exposure.

### 4.2 The effects of Cd on the transcriptomic and metabolic profiles of *Gynostemma pentaphyllum*

Based on the transcriptomics data, 4921 DEGs were detected across the treatment groups (**Figure 2** and **Table S2**). Analysis of DEGs found that they were associated with specific biological pathways, oxidoreductase activity, protein kinase activity and protein phosphorylation, indicating that the *G. pentaphyllum* seedlings could efficiently activate its defense system and induce the expression of stress-related genes to counter act the Cd toxicity it encountered (**Figure S2** and **Table S3**). In prior studies, it was demonstrated that Cd stress exerted a substantial impact on the expression levels of DEGs which were related to amino acid, carbohydrate, and nucleotide biosynthetic pathways in sorghum and *S. nigrum* (Wang et al., 2022; Jiao et al., 2023). These DEGs exhibited marked induction in the biosynthesis of other secondary metabolites, carbohydrate, amino acid and lipid metabolism, as well as signal transduction pathway, which were stimulated in response of *G. pentaphyllum* to Cd stress (**Figure S3**). Earlier studies had demonstrated that the XTH gene family was implicated in the production of hemicellulose in the primary cell walls, potentially serving as an essential mechanism for alleviating Cd toxicity in tobacco and *A. thaliana* (Zhu et al., 2013; Han et al., 2014). Studies have also documented that overexpression of *PtoEXPA12* and *TaEXPA2* in tobacco plants increased the resistance to Cd accumulation (Ren et al., 2018; Zhang et al., 2018). XTHs and EXPAs were significantly expressed after Cd treatment to respond to Cd stress (**Table 1**). In addition, another study showed that XTHs and EXPAs might be candidate genes to regulate cell wall Cd fixation by polysaccharides (Xiao et al., 2020). A greater amount of polysaccharide accumulation was observed in the Cd treatment group (**Figure 1D**). Therefore, we hypothesized that XTHs and EXPAs could potentially play a role in Cd tolerance by fixation of Cd with cell wall polysaccharides. Nevertheless, further investigations are essential to verify this hypothesis. GST is a crucial enzyme involved in enzymatic detoxification, and it plays a vital role in the ROS-scavenging system by catalyzing the interaction of GST with hydrogen peroxide (Strange et al., 2001). Here we also found that two GST genes were significantly up-regulated after Cd treatment (**Table 1**), suggesting that high expression of GST genes in the *G. pentaphyllum* may be involved in Cd detoxification, thereby promoting ROS-scavenging. The LHC family gene LHCB3 was found to be specifically expressed after Pb treatment, and it was able to improve the photosynthesis efficiency of *Trifolium pratense* grown under Pb stress (Meng et al., 2022). Notably, the six LHC family genes were down-regulated under Cd stress, and more specifically so at high Cd concentrations (**Table 1**), when the leaves showed an apparent chlorosis phenotype (**Figure 1A**), which may regulate the photosynthesis of *G. pentaphyllum*.

From the metabolomics data, 126 DAMs were detected across the treatment groups (**Figure 4** and **Table S5**). These DAMs were involved in pyruvate metabolism, TCA cycle, glyoxylate and dicarboxylate metabolism, arginine and proline metabolism, flavone and flavonol biosynthesis pathway, valine, leucine and isoleucine biosynthesis pathway (**Figure S5**), indicating that the *G. pentaphyllum* seedlings could efficiently activate these metabolites as a response mechanism to enhancing the tolerance of Cd. The TCA cycle can enhance energy supplies and elevate the Cd stress-related protein levels by increasing the intracellular carbohydrate content, which ultimately ameliorates Cd toxicity in the roots of *S.nigrum* (Wang et al., 2022). The TCA cycle-related metabolites, citric acid, such as α-ketoglutaric acid, L-(-)-malic acid, succinic acid and fumaric acid, were significantly accumulated after Cd treatment (**Tables S3**, **S4** and **Figure 4D**), implying that they may play a vital role in maintaining energy support balance during Cd stress. Flavonoids, as secondary metabolites, are mainly used to reduce Cd poisoning through chelation and passivation in plants and these can help to confer Cd resistance (Li et al., 2015; Zhu et al., 2020; Jiao et al., 2023). Flavonoids, including luteolin-7-O-(6’’-malonyl) glucoside-5-O-rhamnoside, quercetin-3-O-glucoside (isoquercitrin), quercetin-3-O-galactoside (hyperin), kaempferol-3-O-robinobioside (biorobin) and quercetin-7-O-glucoside were found to be accumulated during Cd treatment (**Figure 4D**), and there must be is involved in the Cd response of *G. pentaphyllum*. Amino acids can effectively chelate metal ions and reduce the toxic effects of Cd on the rice plant, *Noccaea caerulescens* and *N. praecox* (Zemanová et al., 2017; Xue et al., 2022; Kocaman, 2023). The findings of this study suggest that Cd treatment resulted in significant elevations in the levels of L-isoleucine and L-arginine (**Figure 4D**), implying that Cd exposure could stimulate the biosynthesis of these amino acids in *G. pentaphyllum*, potentially enhancing stress resistance.

### 4.3 The effects of Cd on the key metabolic pathways of *Gynostemma pentaphyllum*

Transcriptomics and metabonomics studies have demonstrated that alpha-linolenic acid metabolism plays an important role in tolerance and detoxification to Cd stresses of cotton, rice and fescue, and have identified a significant number of its metabolites and genes that are either induced or inhibited under Cd treatments (Zhu et al., 2018; Zeng et al., 2021; Li et al., 2022a). In this study, a substantial number of DEGs and DAMs were identified that were associated with alpha-linolenic acid metabolism, starch and sucrose metabolism, phenylpropanoid biosynthesis, and ABC transporters as crucial pathways in Cd stress responses in *G. pentaphyllum* seedlings (**Figures 5**–**7**). Moreover, alpha-linolenic acid and 12-OPDA metabolites, as well as ACX, LOX, OPR, and PLA2G genes were identified as the key metabolites and genes in this study (**Figure 6A**), which were related to the production of JA (Ghorbel et al., 2021). JA alleviates Cd toxicity via suppression of Cd uptake and either translocation or reduction of the accumulation of ROS in plants (Lei et al., 2020; Ahmad et al., 2021; Kaushik et al., 2022; Li et al., 2022c). These results suggest that alpha-linolenic acid metabolism was significantly affected by Cd stress, which may have contributed to the impacted key metabolite accumulation and gene expression, thereby promoting JA biosynthesis to alleviate Cd toxicity. Glucose can also alleviate Cd toxicity by enhancing Cd fixation in the cell walls of Arabidopsis roots and sequestering Cd into the vacuoles (Shi et al., 2015). Increased levels of chlorogenic acid in *Kandelia obovata* can reduce Cd and Zn poisoning through its hydroxyl radical scavenging capacity (Chen et al., 2020). It can be speculated that the upregulation of genes involved in the phenylpropanoid biosynthesis pathway together with the increase in chlorogenic acid metabolites is closely related to *G. pentaphyllum* response to Cd stress. The ABC transporter-associated *TaABCC1* and *OsABCC9* genes have been shown to promote Cd compartmentalization in the vacuoles which can enhance Cd tolerance in Cd-tolerant wheat and rice (Yang et al., 2021; Zhang et al., 2023). Our results provide evidence that these key metabolic pathways were significantly responsible for Cd stress, which may have contributed to the impacted key metabolite accumulation and gene expression observed in these conditions.

As crucial transcriptional regulators, WRKY, MYB, and bZIP must play a significant role in the Cd response of plants (Yuan et al., 2018; Li et al., 2023a). Overexpression of *ThDRE1A*, *ThMYC1*, and *ThFEZ* in *T. hispida* decreased the ROS content after Cd treatment by changes in the enzymatic activities of SOD, CAT, and POD (Xie et al., 2023). Overexpression of *PyWRKY75* promoted the absorption and accumulation of Cd, and activated the antioxidant enzymes under Cd stress in poplar (Wu et al., 2022). *PvERF15* positively regulated Cd tolerance by binging to metal response element-binding TF (*PvMTF-1*) (Lin et al., 2017). These findings indicated that TFs are crucial in Cd stress signal transduction, as they interact with various genes associated with Cd uptake, transport, and tolerance, functioning either as transcriptional activators or repressors. Moreover, *AtMYB12* can increase the phenylpropanoid content by activating PAL, C4H, 4CL, and CHS gene expression (Xu et al., 2022). *MeERF72* was found to exert a negative regulatory effect on the expression levels of *MeSuS1* in cassava (Liu et al., 2018). *PuERF12* and *PuMYB44* were identified as candidate TFs that may regulate the expression of *PuLOX2S* in Nanguo pears (Zhang et al., 2022). In this study, MYB, MYB_related, ERF, GRAS, bZIP, WRKY and bHLH could effectively interact with phenylpropanoid biosynthesis, starch and sucrose metabolism and alpha-linolenic acid metabolism pathway genes (**Figure 8**), suggesting these TFs impact on the accumulation of these metabolites, thereby contributing to the *G. pentaphyllum* response to Cd stress.

**Figure 9:**
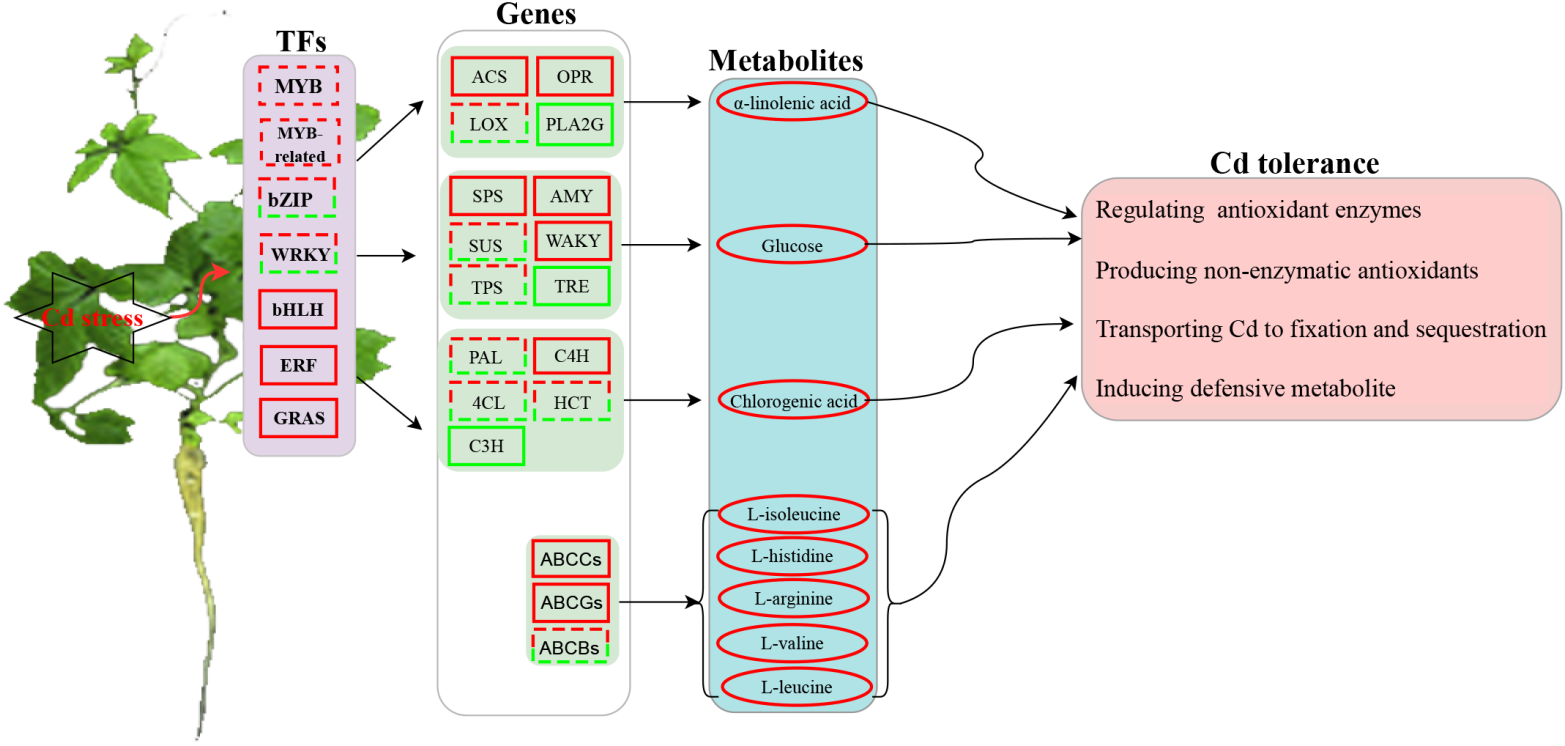
A predicted model of transcription- and metabolism-driven mechanisms in *G. pentaphyllum* in response to Cd stress. The square represents the TFs (transcription factors) or genes, and the ellipse represents the metabolites (solid line red: genes up-regulated or metabolites accumulation; solid line green: genes down-regulated; dashed red-green lines: some genes up-regulated or down-regulated).

A possible model of transcription- and metabolism-driven Cd response mechanisms in *G. pentaphyllum* is shown in **Figure 9**. Cd could either activate or repress the expression of TFs, including MYB, MYB_related, ERF, GRAS, bZIP, WRKY and bHLH. In addition, phenylpropanoid biosynthesis, starch, and sucrose metabolism, and alpha-linolenic acid metabolism pathway genes were activated and these increased the levels of alpha-linolenic acid, glucose, and chlorogenic acid metabolites. The up-regulated genes such as ABCCs and ABCGs upon Cd application highly activated the synthesis or transport of ABC transporter-related metabolites (L-isoleucine, L-histidine, L-arginine, L-valine, and L-leucine). Taken together, the TFs, ABC transporter genes, and metabolites (i.e. JA, glucose and phenylpropanoid) might all play important roles in *G. pentaphyllum* response to Cd by regulating the activities of antioxidant enzymes and the production of non-enzymatic antioxidants. These would work together to transport, fix, and sequester Cd, thereby inducing the defensive metabolite response for *G. pentaphyllum* to adapt better to the Cd stress. These results provide groundwork for comprehending the Cd response mechanisms in *G. pentaphyllum*. Nevertheless, the functions as well as the biochemical mechanisms of how these TFs, genes, and metabolites perform their tasks require further study.

## 5 Conclusion

Omics analysis was used to explore the Cd stress response and tolerance mechanism of *G. pentaphyllum*. This study suggests that *G. pentaphyllum* seedlings could significantly activate the POD, CAT and APX enzymatic activities as well as increase the proline and polysaccharide content in response to Cd stress. Transcriptomics analysis revealed that 4921 DEGs responded to Cd stress, and these involved secondary metabolites, carbohydrate metabolism, amino acid metabolism, lipid metabolism, and signal transduction pathways. By using metabolomics analysis, a total of 126 DAMs were identified, and citric acid, flavonoid (diosmetin-7-O-rutinoside, diosmetin-7-O-galactoside and 6-C-methyl kaempferol-3-glucoside) and the amino acids, L-isoleucine and L-arginine were significantly increased after Cd treatment. A large number of candidate genes and metabolites were also identified in alpha-linolenic acid metabolism, starch and sucrose metabolism, phenylpropanoid biosynthesis, and ABC transporters. In addition, MYB, MYB_related, ERF, GRAS, bZIP, WRKY and bHLH TFs may regulate the expression of genes and metabolites accumulation, and all these processes appeared to contribute to *G. pentaphyllum* response to Cd stress.

## Data availability statement

The datasets presented in this study can be found in online repositories. The names of the repository/repositories and accession number(s) can be found in the article/**Supplementary Material**.

## Author contributions

YZ: Conceptualization, Data curation, Writing – original draft, Writing – review & editing. LXY: Data Curation, Methodology, Visualization, Investigation. XH: Methodology, Visualization, Investigation. YL: Visualization, Investigation. CW: Visualization, Investigation. QH: Data Curation. LYY: Supervision, Funding acquisition, Resource mobilization, Validation. CP: Project administration, Funding acquisition, Writing – review & editing.
