# Supplementary material for: Transcriptomics and metabolomics association analysis revealed the responses of Gynostemma pentaphyllum to cadmium
Source: Front Plant Sci. 2023 Oct 9;14:1265971. doi: 10.3389/fpls.2023.1265971 (PMC10591085; doi:10.3389/fpls.2023.1265971)
Supplement: Supplementary file 1 [file DataSheet_1.docx]

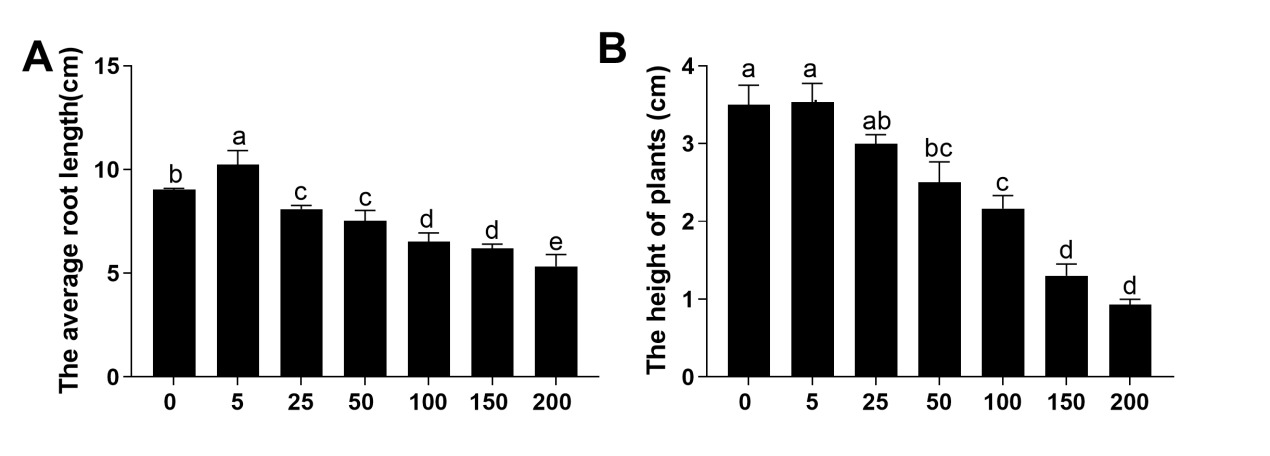


Fig.S1 Effects of treatment with different concentrations of CdCl_2_ on root length and height in *G. pentaphyllum* seedlings.


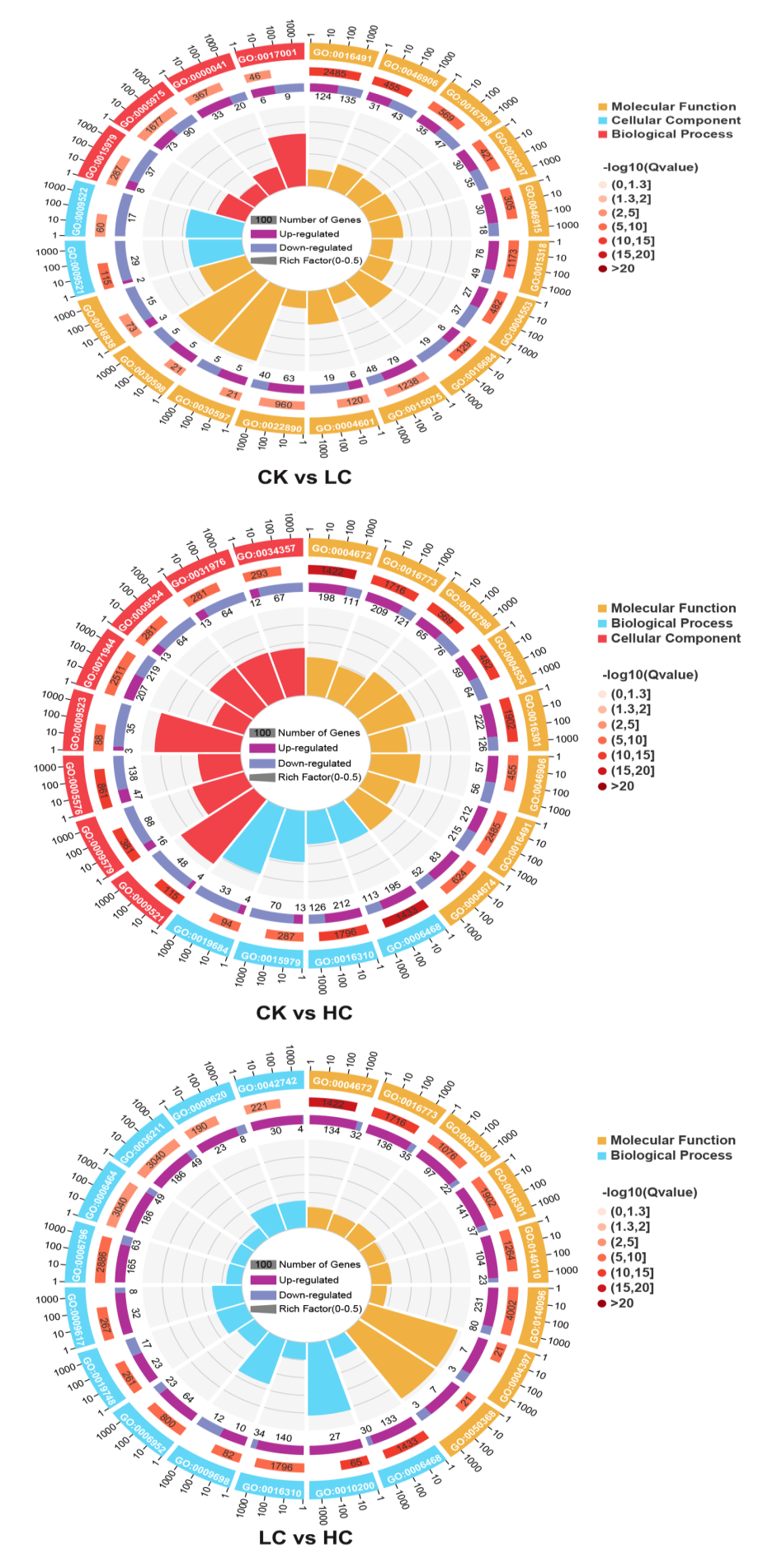


Fig.S2 The Gene Ontology (GO) enrichment analysis of DEGs among the different treatment groups.
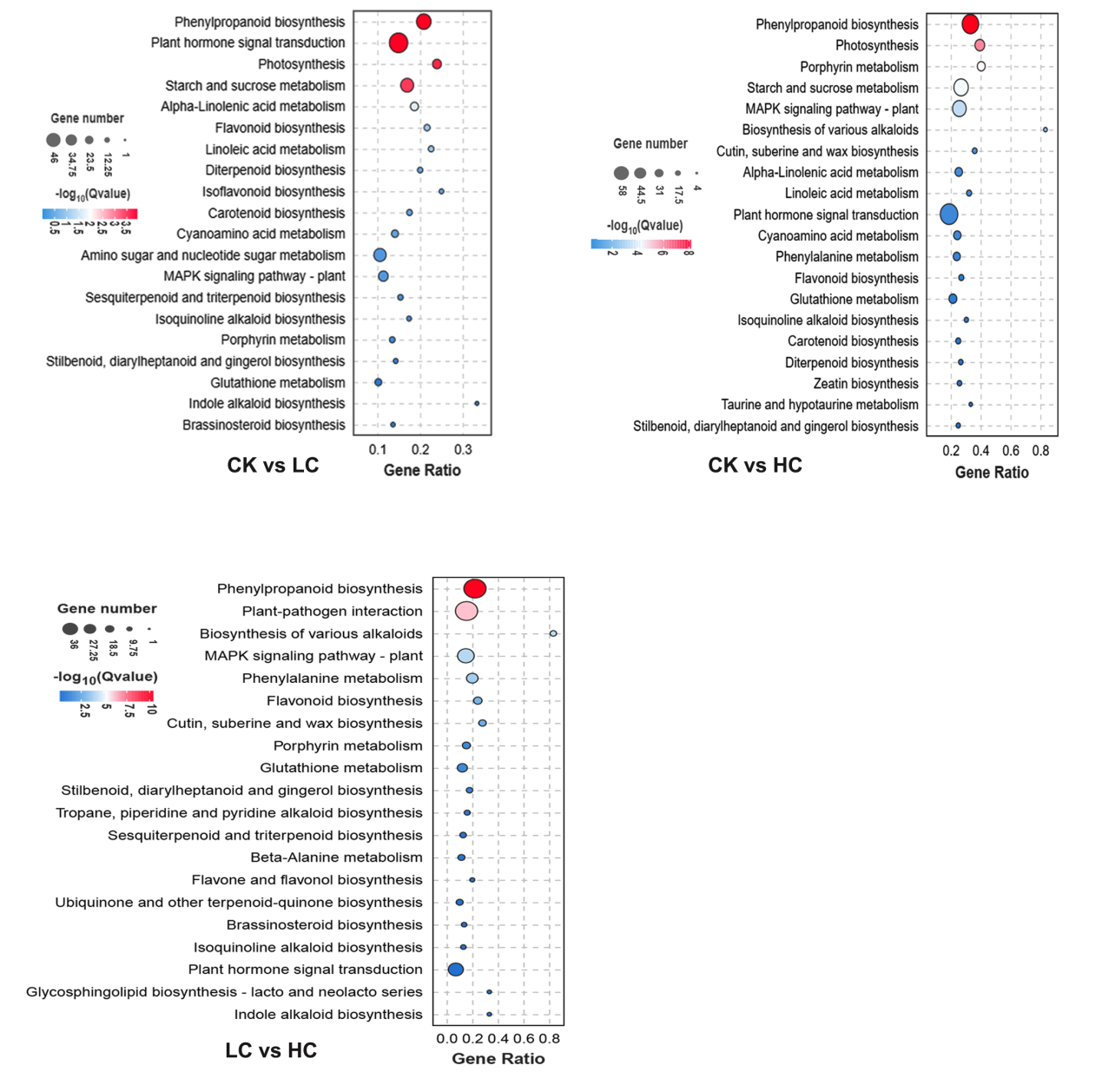


Fig.S3 The KEGG enrichment analysis of DEGs among the different treatment groups.
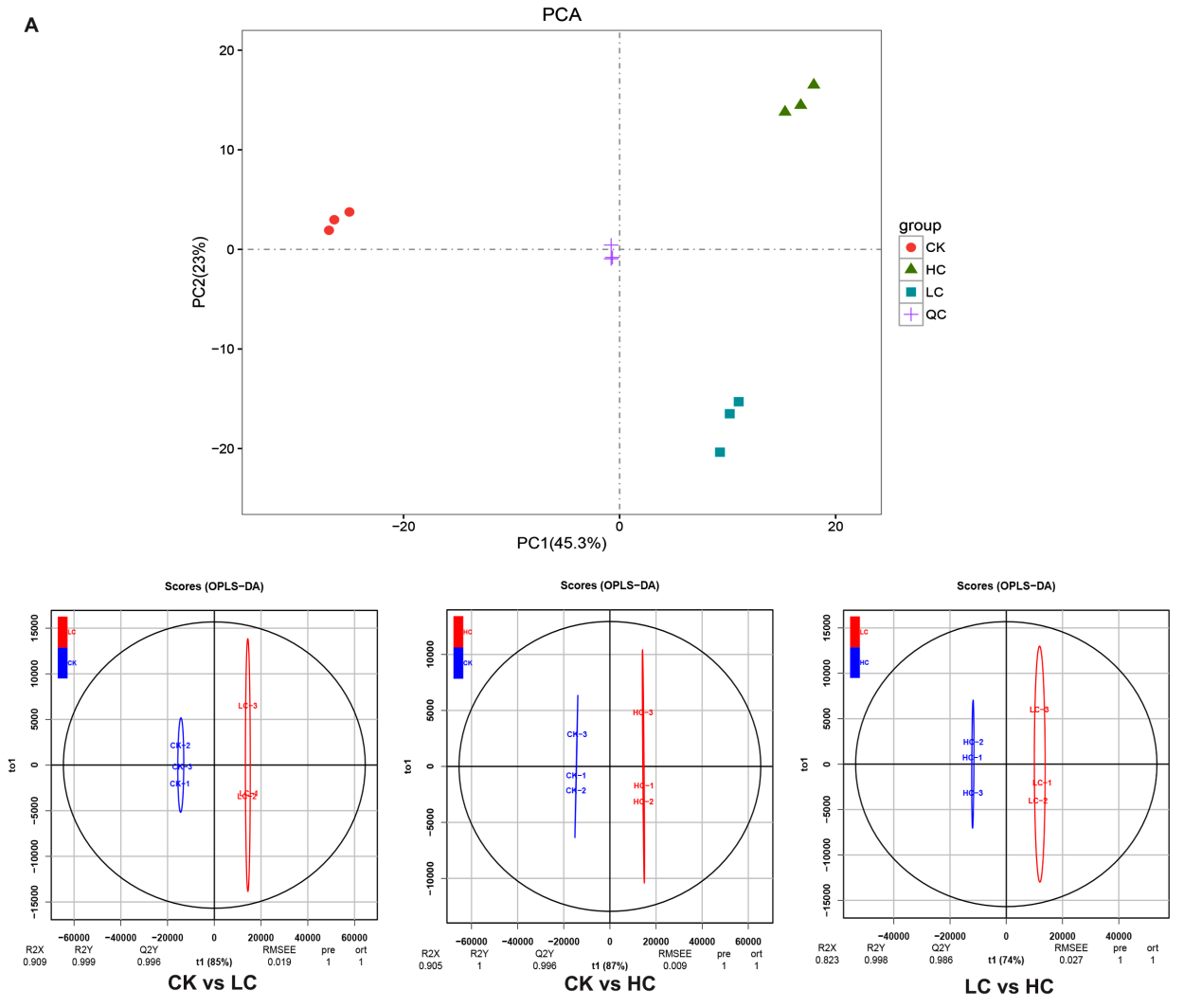


Fig.S4 The PCA and OPLS-DA score plot analysis of different treatment groups.


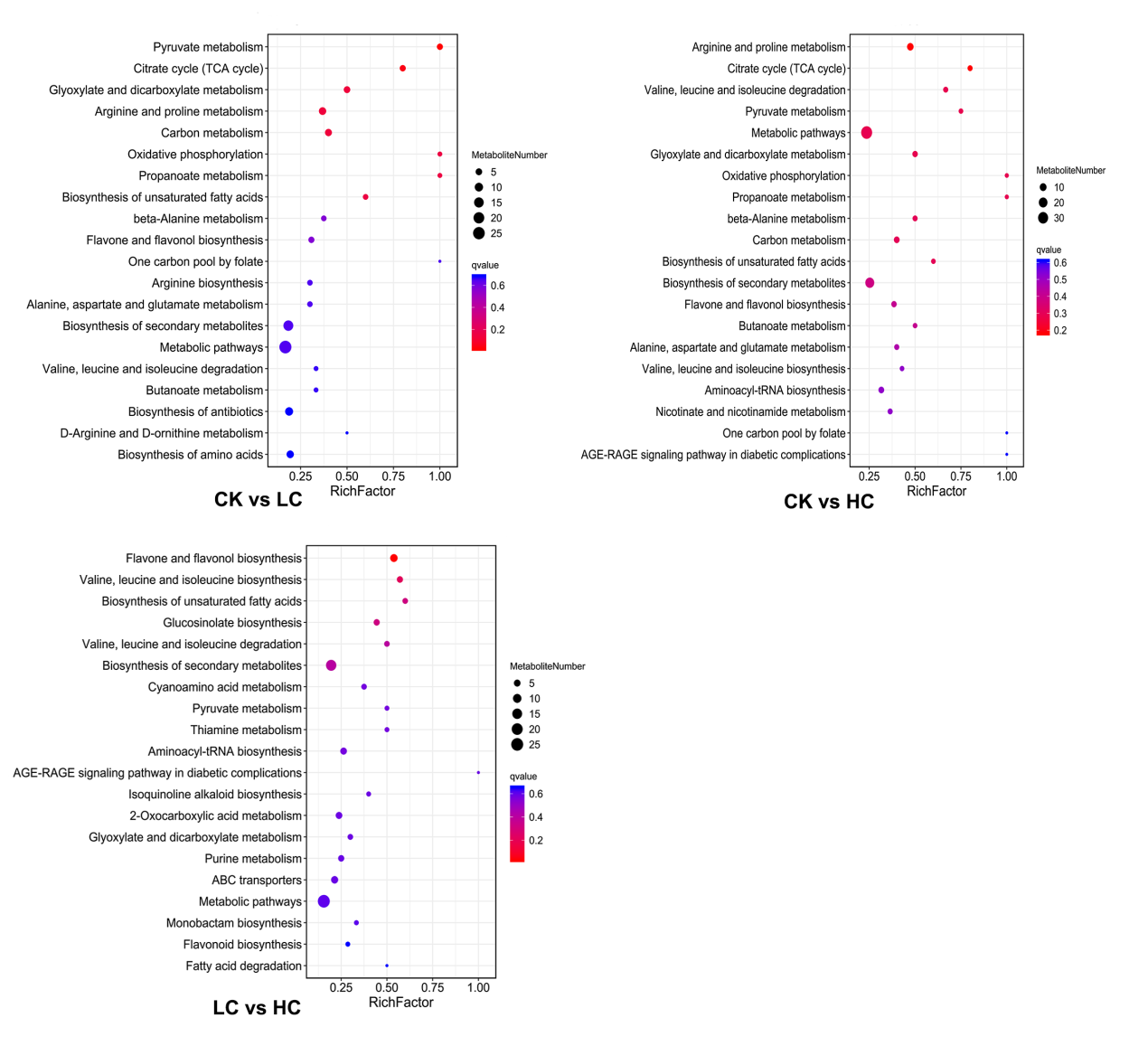


Fig.S5 The KEGG enrichment analysis of DAMs among the different treatment groups.


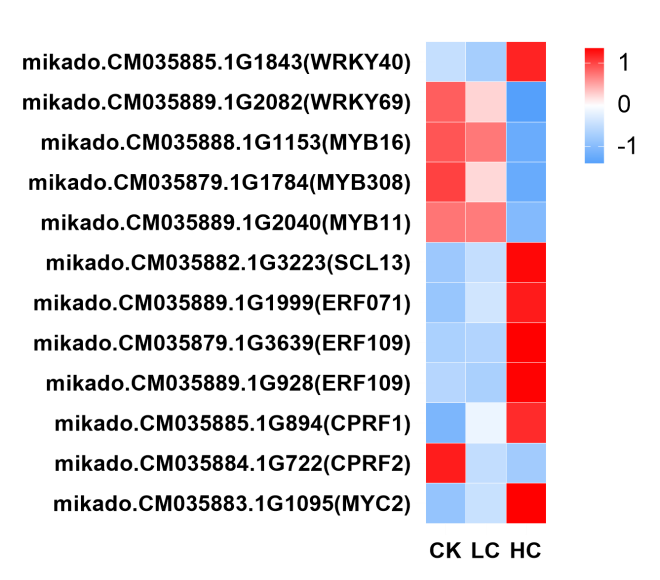


Fig.S6 The heatmap analysis of TFs compared between different groups.


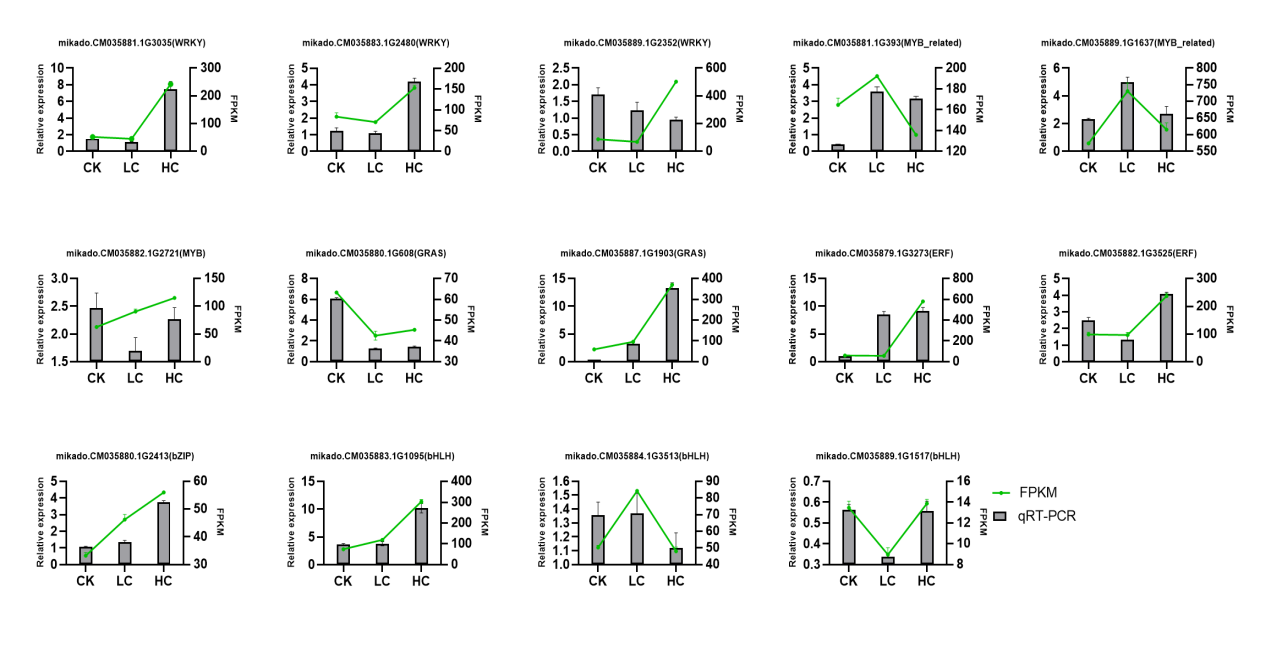


Fig.S7 The relative expression levels of candidate genes. The histogram indicates gene expression data from qRT-PCR. The line chart indicates FPKM from transcriptomic data. Data are presented as means ± standard deviation (n = 3).
